# The comparison of the technical parameters in endotracheal intubation devices: the Cmac, the Vividtrac, the McGrath Mac and the Kingvision

**DOI:** 10.1007/s10877-015-9727-2

**Published:** 2015-06-29

**Authors:** Marcin Cierniak, Dariusz Timler, Andrzej Wieczorek, Przemyslaw Sekalski, Natalia Borkowska, Tomasz Gaszynski

**Affiliations:** 1 Department of Emergency Medicine and Disaster Medicine, Barlicki University Hospital, Medical University of Lodz, Ul. Kopcinskiego 22, 90-153 Lodz, Poland; 2 Department of Anesthesiology and Intensive Therapy, Medical University of Lodz, Lodz, Poland; 3 Department of Microelectronics and Computer Science, IT Centre, Lodz University of Technology, Lodz, Poland

**Keywords:** Cmac D-blade, McGrath Mac, Vividtrac, Kingvision

## Abstract

Currently, there are plenty of videolaryngoscopes that appear on the market. They have different specifications. Some of these features favor the fact that they are more suited for educational purposes of future operators and others can be characterized with an excellent clinical use. In this study we compared four types of videolaryngoscopes. The aim of the study was to compare the technical specifications of the above-mentioned devices for usefulness in clinical practice and correlate these parameters with the subjective evaluation of these videolaryngoscopes usage performed in practice by an experienced medical staff. All devices considered in this study participated in another multicenter clinical study on the basis of which we completed the subjective evaluation of the operators. In order to examine the technical parameters of the equipment we established the cooperation with the Department of Microelectronics at Technical University of Lodz. Mechanical and optical parameters and the endoscopic tube current were taken into consideration. The C-MAC has a camera with the widest viewing angle (the OX axis—63.1, the axis OY—47.8), which in combination with the largest diagonal size of the display enables the operator to see the details relevant to clinical practice. It has also the strongest lamp intensity of the devices mentioned in this comparison (7800 Lx). In comparison of the clinical use in almost all compared parameters the Cmac D-blade is a winner, although for clinical education purpose we consider the Vividtrac a better device.

## Introduction

In recent years, with the advent of videolaryngoscopes on the market, the approach to advanced managing of the upper respiratory tract has changed. Videolaryngoscopes gained popularity as a device that can significantly help the operator to visualize the entrance to the larynx in difficult intubation even if the patient was graded by Cormack–Lehane scale with the use of traditional Macintosh laryngoscope achieved the third and fourth degree [1]. Moreover videolaryngoscopes are described as devices helping to reduce peri-intubation complications for instance by reducing the amount of intubation attempts and shortening its time [2, 3]. Videolaryngoscopes use video camera technology and fiber technologies. They are used both for operating theatre and intensive care units. Due to technologies used in these instruments the effectiveness of intubation increased [4–6]. There are many factors in favor of using videolaryngoscopes also in pre-hospital care where the problem of difficult airway is quite common. Many times, in the pre-hospital environment we have to deal with the difficult conditions prevailing at the scene. Videolaryngoscopes take many kinds of shapes and sizes.

**Fig. 1:**
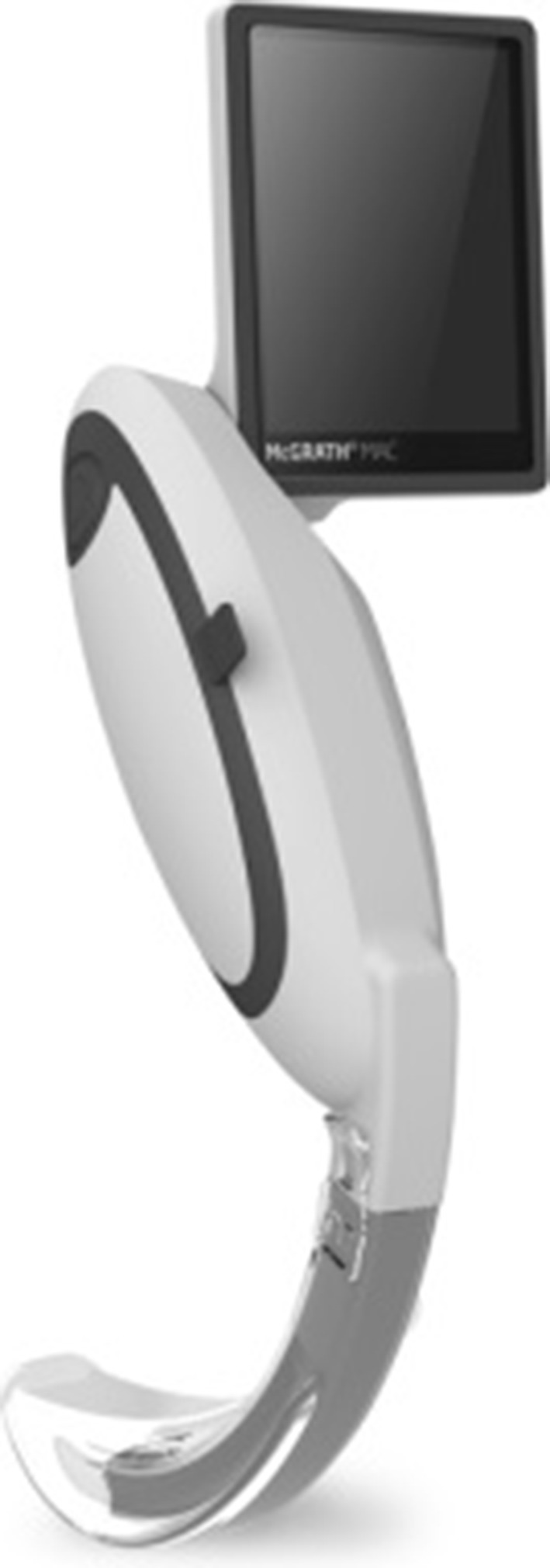
McGrath Mac videolaryngoscope. *Source* http://www.covidien.com/rms/products/video-laryngoscopy/mcgrath-video-laryngoscope
- Kingvision (Fig. 2): The videolaryngoscope with an LCD screen rigidly attached. The device is powered by three AAA batteries. It has removable plastic disposable blades of the same size. Each of the blades has a track where the endotracheal tube can be provided [7].

In our study, we decided to assess four videolaryngoscopes in terms of their technical parameters and describe subjective impressions from using these appliances in clinical practice. The opinion given by the operators using these devices has been collected in the course of different clinical trial concerning intubation where all of these four appliances were used. This was a multicenter clinical trial including 153 patients, and it was carried out to monitor postintubation injuries. The operators were always specialist doctors of emergency medicine or anesthesiology with a minimum of 15 years of work experience. To control the technical parameters we invited the Department of Microelectronics at the Technical University of Lodz. They examined the above mentioned devices at an angle of technical parameters at our request. In this article we will try to compare the examined technical parameters to the authors’ subjective opinions in order to indicate the best solution for difficult intubation providing the best field of vision, good lighting, contrast and quality of the image itself. However, at the beginning, these devices will be described briefly (expanded description in Sect. 3):

- McGrath Mac (Fig. 1): A videolaryngoscope with the LCD screen enabling vari-angle position. It has removable disposable plastic blades of different sizes that match one device and it includes a 250-min battery with a minute-by-minute on-screen.

**Fig. 2:**
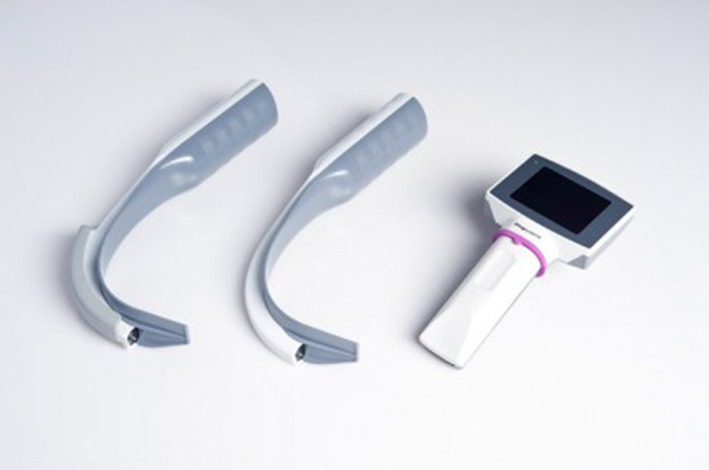
Kingvision videolaryngoscope. *Source* http://www.owntheairway.com/king-video-laryngoscopes/
- Vividtrac (Fig. 3): The image from the videolaryngoscope is displayed on the connected device such as a laptop or a tablet and for the sake of proper operation vividvision should be used. It is powered through the USB port of the device. It has a track for the endotracheal tube. There is no need to tilt a head during videolaryngoscopy what is perceived as an advantage by the manufacturer. Vividtrac is one size only—adult 3.

**Fig. 3:**
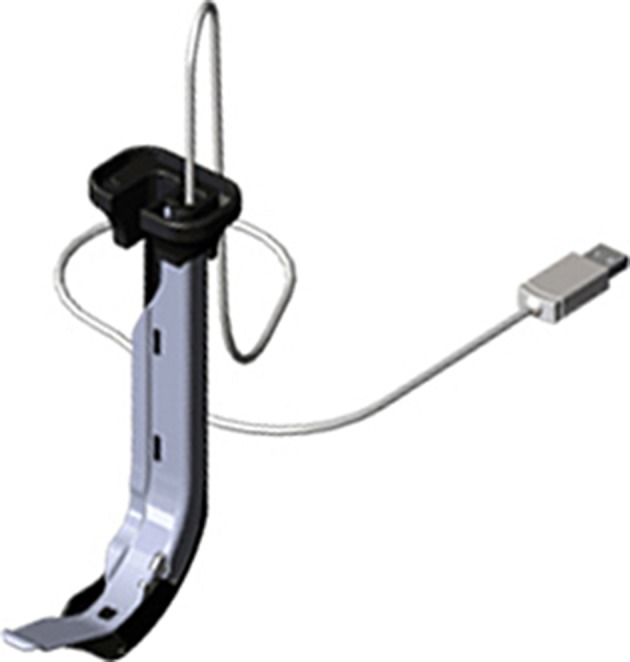
Vividtrac. *Source* http://mercurymed.com/catalog2/index.php?type=87
- Cmac Dblade (Fig. 4): The device produced by Storc. It is equipped with an internal rechargeable battery. This videolaryngoscope has buttons to take screenshots. Images are stored in an easily accessible memory card. The manufacturer placed the device in a convenient carrying case protecting it against external influences. The laryngoscope has a one-size blade [8, 9].

**Fig. 4:**
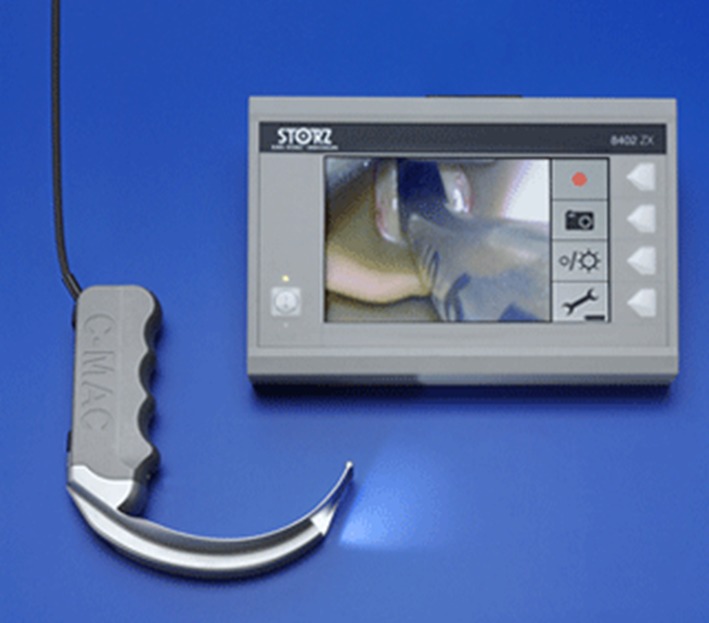
Cmac D-blade. *Source* http://www.medgadget.com/2011/01/dblade_from_karl_storz_improves_view_in_difficult_airway.html

## Materials and methods

As already mentioned, all the devices considered in this study participated in a multicenter clinical study that monitored and documented postintubation injuries. The photographs are taken from that study (Fig. 12). As mentioned previously, to examine the technical parameters of the devices we established cooperation with the Department of Microelectonics at Technical University of Lodz. The person responsible for comapartion of technical parameters was Przemysław Sękalski from Technical University of Lodz and the rest five authors were responsible for the device clinical evaluation. Such information was placed in original article too. The methodology of studying certain parameters is described below. Mechanical and optical parameters and measuring the intensity of the endoscopy light were parameters taken into account.

### Mechanical parameters of the devices

The distance between the camera and the end of the laryngoscope blade. The measurement was made by the caliper. Owing to rounding the error of measurement should be at the level of ±1 mm. Measurement error is also influenced by the fact that the camera is hidden in the device and it is impossible to measure the distance between the sensor and the distal blade tip. The only exception is the VividTrack VT-A100 with the camera lens on top of the device. It was assumed that the distance is measured from the housing, wherein the camera is attached to the end of the device.

### Measurements of endoscopic light intensity

The measurement of light intensity was performed using the lux meter LX-105 by Lutron Electronic Enterprise having been awarded ISO-9001, CE and IEC1010 certificates. The device can measure the intensity of light coming from different sources, in particular: daylight, incandescent, fluorescent and mercury light. Unfortunately, the model cannot measure the intensity of one light temperature, as it is in the semiconductor used in the sources mentioned above. Therefore the measurement is qualitative rather than quantitative. Measurements were made in a dark room where the only source of light was the endoscope. Measurements were made with the fully equipped endoscopes. Each time the end of the device was applied to the lux meter. In the case of the McGrath the measurement was performed using a range of 0–1999 Lx, for the other devices in the range of 2000–19,990 Lx.

### The optical parameters of the devices

**Fig. 5:**
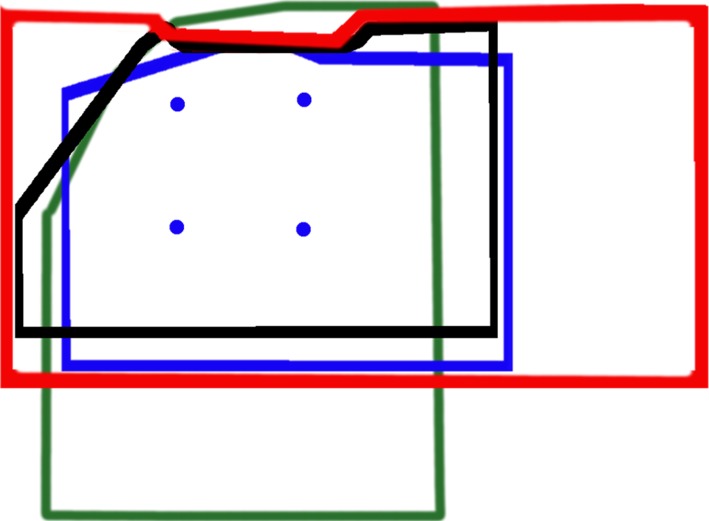
The field of view of each device. VividTrack VT-A100 (*red*), C-MAC 8401 ZX (*black*), KingVision (*blue*), McGRATH MAC (*green*). The *dots* represent corners of a *square* with a side measuring 1 cm. The imaging plane at the distal blade tip

The measurement was performed by the graph paper with the tip of the blade and calculating the area seen in the display. The image was observed in the XY system. The visual fields of particular devices were also superimposed on each other to compare the range of visibility (Fig. 5).

## Results

The results section is divided into subjective impressions of the machine operator, which were noted during the research mentioned above, and the section concerning technical parameters.

Operators’ practical impressions concerning the devices:

- Kingvision: In case of this device one of the disadvantages could be the rigid fixation of the LCD screen and a relatively large cross-section of the device which may bring some difficulties during the difficult intubation in a patient with slight oral dilation. The lack of opportunity to rotate the LCD screen causes that the operator focusing on the correct laryngoscope’s insertion in the mouth cannot see the screen for the first phase of intubation. Only after the insertion of the laryngoscope and passing the patient’s uvula, the angle between the eye of the operator and the screen is reduced so that the operator can effectively evaluate what is seen on the screen. It is also worth mentioning that the power supplied by three AAA batteries is not sufficient for a long-term use.
- McGrath Mac: In our opinion this device is relatively handy. The great advantage is that it has removable, disposable plastic blades, which reduces the risk of scratching the patient’s teeth. The screen provides a relatively wide field of view in the vertical direction. During visualization of the epiglottis, the patient’s uvula is still in the field of view. Unfortunately, due to technical reasons (no external connection to a computer), it was impossible, as in the case of the Kingvision, to take a screenshot. We have tried to take LCD screen’s pictures using a camera, but the interference alters the image so that it is not suitable to be shown in the article. The movable LCD screen is a very useful thing. The operator depending on the existing situation can freely change the angle of the screen. Considering that the power supply is a battery that can only be purchased from the manufacturer because its shape is unique and fits only for this unit, it can be perceived as a disadvantage.
- Vividtrac: From the point of view of the operator, the device is very convenient to use. Comparing to the previous devices, when it is used with a laptop, there is a very large screen. The disadvantage of this device is that a strong flash causes the mucous membranes overexposure. Furthermore, the vividvision program moves hue towards red with the result that sometimes the operator may feel that the mucosa is congested. The drawback of this device was fogging of the camera which was hard to clean and made the videolaryngoscopy difficult to continue. A significant advantage from the educational point of view is the ability to take pictures while using the vividvision program. The device requires no power source. The device is powered by the equipment to which it is connected through the USB interface.
- Cmac D-blade: This device has a well-chosen color balance therefore the operator can see more details in the image. The LCD screen is not in large size, but sufficient to identify all structures of the airways in the field of view. Recording buttons and taking pictures buttons are both in the device’s screen and on the laryngoscope handle what facilitates taking pictures. From the educational point of view, it is a very big advantage because the images are stored in a universal format in the memory card and then can be presented to students in any medium. The handle is ergonomically designed. One size of the laryngoscope blade can be a disadvantage. In our opinion, in terms of powering, it is the best device, because the internal battery can be recharged by line voltage. There is also a unique protective bag attached to the device by the manufacturer which perfectly harmonizes with the device and protects it from any external factors.

The results concerning technical parameters are presented below:

- The mechanical parameters of the devices
- The measurement of the intensity of the endoscopic light

It is clear that both devices: the KingVision and the C-MAC 8401 ZX have stronger light source which results in better contrast of the observed images. The large difference in light intensity readings is due to the fact that the distance between the distal blade tip and the source of light is two times higher in the case of the McGrath than in the other two devices. The intensity of light varies 1/r^2^ which in the case of a doubling of the distance results in a fourfold reduction in light intensity. Taking into account the amendment related to the length of the McGrath blade the intensity of the light source is still twice or three times worse than in the other two devices. Despite the fact that the VividTrack VT-A100 has the same blade’s length as the McGrath MAC, the intensity of light is three times stronger in case of this device what is reflected in the quality of the observed image (Tables 1, 2).

**Table 1:** The distance between the camera and the distal blade tip

	McGrath MAC	KingVision	VividTrack VT-A100	C-MAC 8401 ZX
The distance between the camera and the distal blade tip (mm)	60	34	60	35

**Table 2:** Individual values of the intensity of the endoscopic light

	McGrath MAC	KingVision	VividTrack VT-A100	C-MAC 8401 ZX
C mercury (Lx)	540	6800	1900	8000
L incandescent (Lx)	620	6600	1880	6600
F fluorescent (Lx)	540	5500	1830	7200
S daylight (Lx)	570	6000	1860	7800
Lamp type		White led		LED

- The optical parameters of the devices

The McGrath MAC device as the sole has a display in a vertical position. The other two devices have horizontal displays (Table 3).

**Table 3:** Optical parameters

Screen type	McGrath MAC	KingVision	VividTrack VT-A100	C-MAC 8401 ZX
	Vertical	Horizontal	Horizontal	Horizontal
Area observed in the OX axis (mm)	30	34	58	43
Area observed in the OY axis (mm)	41	24	28	23
The virtual image of the OY axis (mm)	44	26	45	31
The viewing angle of the OX axis (°)	28.1	51.8	51.6	63.1
The viewing angle of the OY axis (°)	40.3	40.8	41.1	47.8
Reference to the best result for the OX axis (%)	44	82	82	100
Reference to the best result for the OY axis (%)	84	85	86	100

The computations were made by calculating the arctg (0.5∗ distance observed in the OX axis/distance from the camera). To calculate the OY axis the virtual image was used due to the blade which covers a part of the image captured by the camera.

It is clearly visible, that the C-MAC 8401 ZX has a camera with the widest viewing angle, which in combination with the largest diagonal size of the display gives you the opportunity to see the details crucial in the clinical practice. However, due to the camera resolution and optics this device does not ensure the best image quality (Table 4).

**Table 4:** Screen parametres

	McGrath MAC	KingVision	VividTrack VT-A100	C-MAC 8401 ZX
Screen resolution (px)	b.d.	320 × 240 QVGA	n.d.	800 × 480
Screen technology	LED	OLED	n.d.	LCD
Size of the screen (inch)	2.5	2.4	n.d.	7.0
Recording speed (fps)	b.d.	30	30	12
Camera resolution (px)	b.d.	640 × 480 VGA	640 × 480 VGA	320 × 240 QVGA
Sensor technology	CMOS	CMOS	CMOS	CMOS
Pixel density [resolution (px/cm PPCM)]	b.d.	187	109	75

Resolution of more than 200 pixels per inch is invisible to the naked eye. The value of resolution below 100 pixels means that the details are omitted. In the case of the C-MAC the camera resolution is four times worse than in other solutions. However, if the VGA camera is used in the C-MAC, the pixel density will be 150 PPCM. In case of this device the interpolation must be used in view of the screen resolution which is twice higher than the resolution of the sensor.

- The field of view

The range of the observed images in different devices is presented below. The figure renders the field of view of all four devices on the assumption that the imaging plane is at the distal blade tip. Images were scaled so that the size of a square of a side measuring 1 cm (indicated by dots) was identical in each area.

It is obvious that the devices with a longer blade have a larger field of view. But we must remember that it is related to a lower image resolution calculated as the number of px/cm^2^. This can be observed while comparing for example the KingVision and the VividTrack VT-A100 (Fig. 5).

The VividTrack VT-A100 does not have a built-in screen. It should be connected to the computer via USB interface. It is seen as a webcam with VGA resolution (640 × 480 px).

**Fig. 6:**
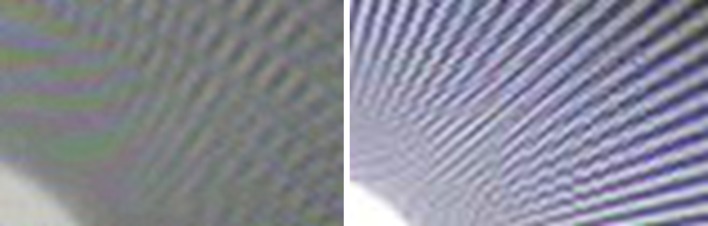
The comparison of test images recorded with the C-MAC (*left*) and the VividTrack (*right*) at a magnification of ×16

**Fig. 7:**
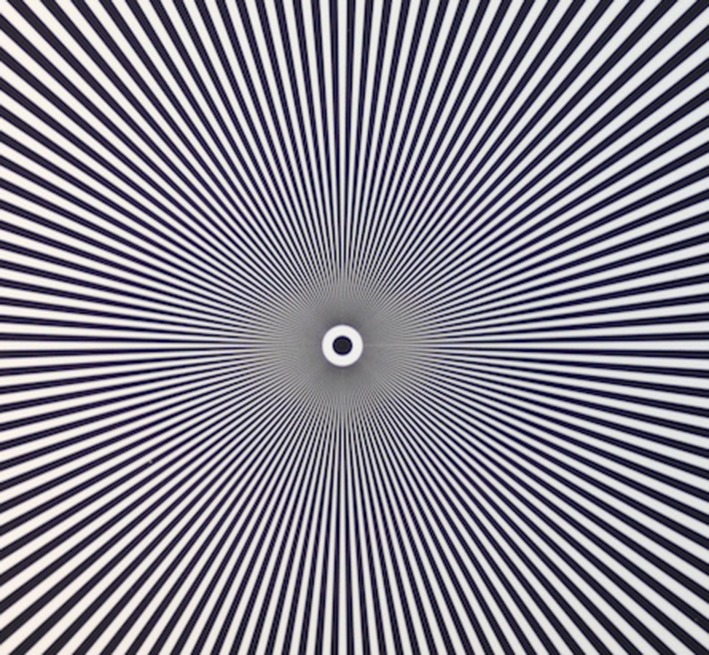
Test pattern

**Fig. 8:**
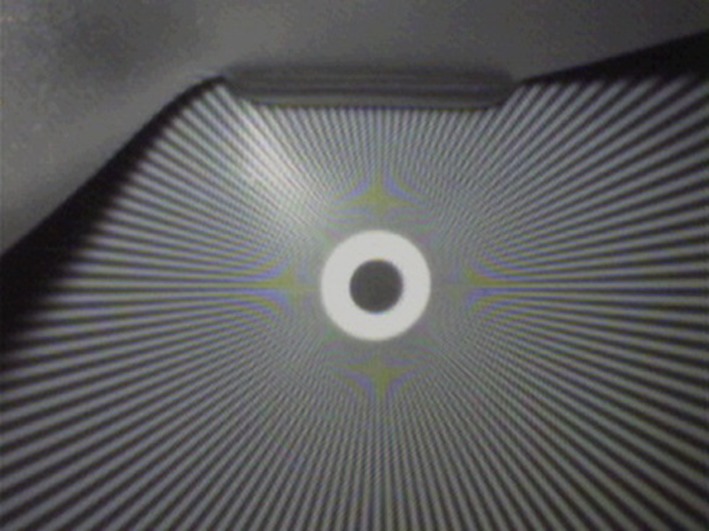
Test image of the C-MAC

The C-MAC 8401 ZX has an unusual display of 800 × 480 while the side menu is 160 × 480 pixels [8]. Therefore the effective image observed by the camera is 640 × 480 px (VGA). There is information that the camera resolution is only 320 × 240 px QVGA. This is evident in the tested images and while comparing the observed image of the VividTrack VT-A100 and the C-MAC 8401 ZX. The McGrath MAC and the KingVision were not compared, due to their inability to record the native image. However, the subjective assessment indicates, that while in case of the KingVision lines can be seen near the center of the control image, the McGrath has a very strong artifacts (moiré pattern) and therefore the image is not sharp (Figs. 6, 7, 8).

**Fig. 9:**
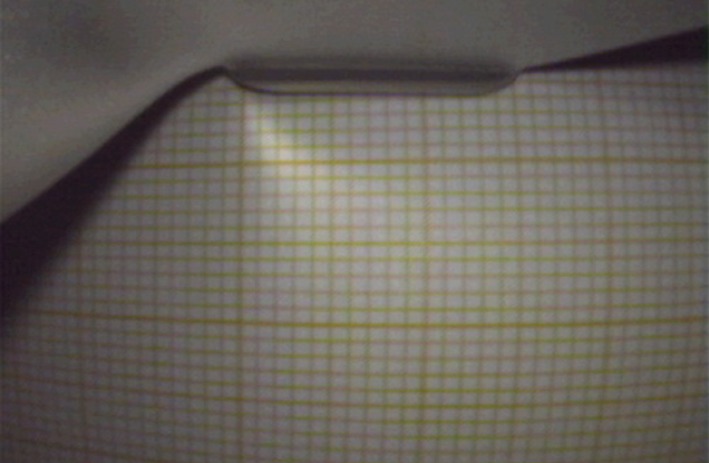
Test image of the C-MAC

**Fig. 10:**
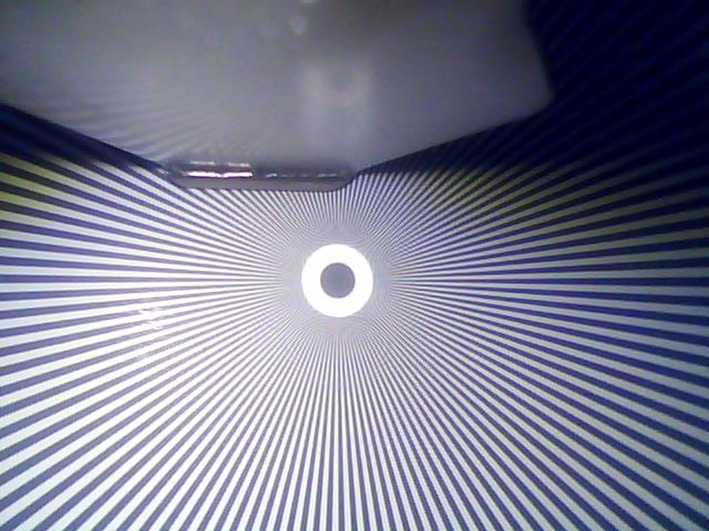
Test image of the VividTrack

The moiré pattern is clearly visible in the form of repeating patterns around a central point. This effect arises when the size of the line is similar to the resolution of the sensor. When objects (here, the line width) are smaller than a single pixel can record, the values are averaged and as in the case mentioned above it revealed as gray (Figs. 9, 10).

**Fig. 11:**
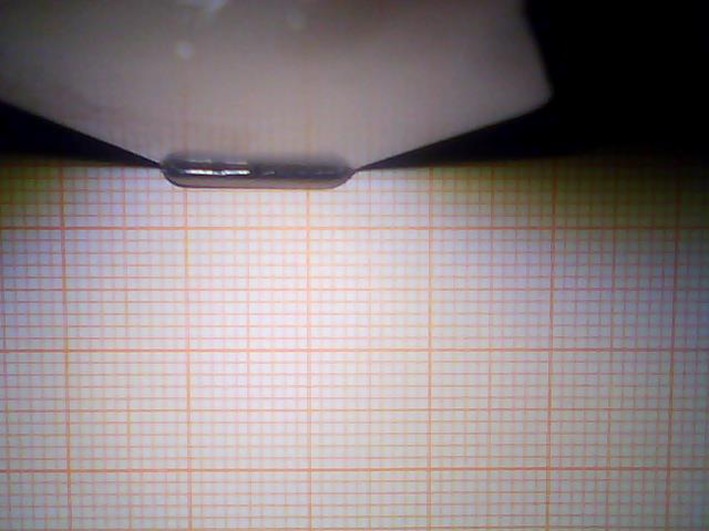
Test image of the VividTrack

However, the moiré pattern also occurs here, it occurs for points located closer to the center of the image. This means that the sensor resolution is higher (2× compared to the C-MAC), and that the adjacent pixels do not have crosstalk (improved selectivity). We can also observe that the picture is more contrast and the colors are more vivid (Fig. 11).

It is visible that the top photo is overexposed, which negatively affects the usefulness of the image (Table 5).

**Table 5:** Fare of each device (in PLN)

	McGrath MAC	KingVision	VividTrack VT-A100	C-MAC 8401 ZX
Fare	26,000	8000	500	29,000

## Discussion

**Fig. 12:**
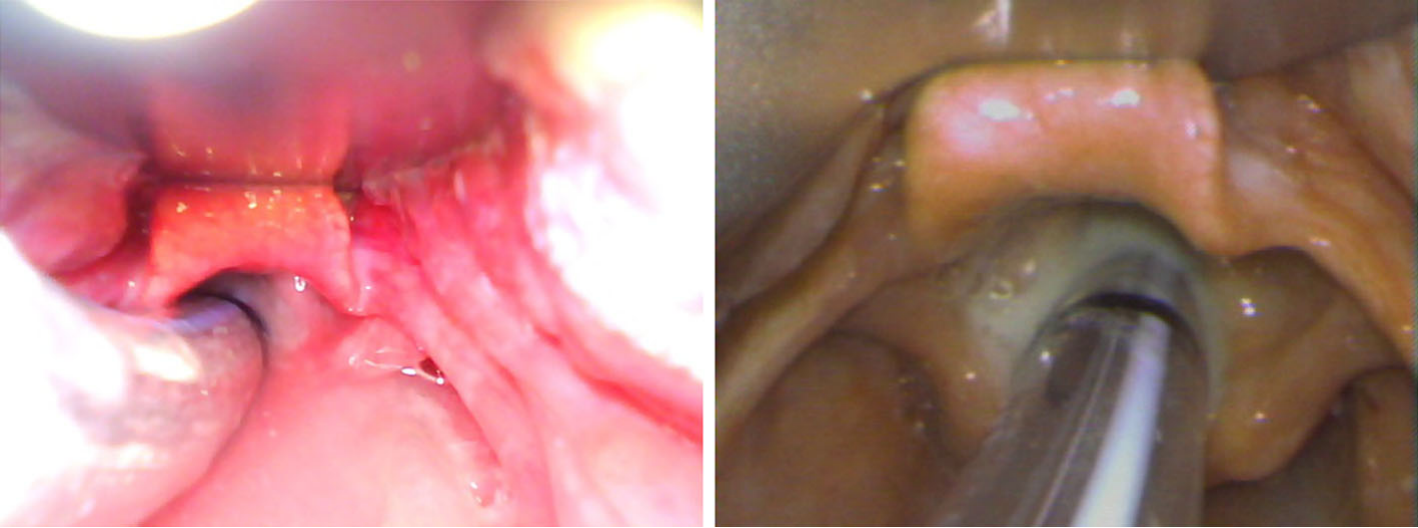
The comparison of the images in the same patient. *Left* image captured using the VividTrack, *right* one through the C-MAC
- Cmac: According to the presented literature this device exceeds almost every videolaryngoscopes in the individual rankings. The authors emphasize a shorter duration of intubation, less possibility of complications and high intubation performance indicators [15–17]. In our study, this device was found to have definitely the strongest endoscopic light (Table 2). It has a camera with the widest viewing angle, which in combination with the largest diagonal size of the display enables the operator to see the details relevant to clinical practice. It also affects the color balance, which is excellent and the images are not overexposed (Fig. 12). In our opinion, this device is very convenient to use and due to the attached protective bag it is suitable for rapid transport and almost immediate use. Owing to an easily accessible memory card which stores the pictures and videos, it is ideal for research and educational purposes. This device wins in almost every test conducted in this study. In the opinion of the operators who had held it in hands, the Cmac is definitely the best device in this comparison. In the literature, there are no data comparing simultaneously four videolaryngoscopes, but in the individual rankings [15–20] the Cmac is also shown as the best device to manage the difficult airway in wide range of patients.

The discussion section is also divided into four subsections describing each device:

- McGrath Mac: In the literature, there are some reports on the effectiveness of the laryngoscope, both in clinical practice and in the case of young medical students training [10–12]. There are also some deficiencies of this device mentioned. In Ray’s study [10] it was discovered that students in spite of the noticeable increase of the field of view, had a problem with placing the endotracheal tube in the trachea. The operators did not notice this drawback in our study. Comparing the technical parameters in our study (Table 2) we find that of all comparable devices the McGrath has definitely the weakest endoscopic lamp. Its field of view (Fig. 5) extends in the vertical axis, ensuring good visibility, but the question whether it is needed has to be answered. Seeing the entrance to the larynx, still in accordance with the above, we have patient’s uvula in the field of view. This feature of the device is not negative, but it also does not bring any benefits.
- Kingvision: Searching the literature one can find very few reports concerning the effectiveness of this device in both clinical practice and in the form of training for students. Most of the reports came from Japan [13, 14] They point to the fact that the device is easier to use in the case of difficult intubation than standard Macintosh blades, and that it provides a good field of view. Our research shows that the Kingvision has a very strong endoscopic lamp (Table 2), which definitely brightens the observation area well. Unfortunately this device compared to others, cannot be distinguished by a wide field of view (Fig. 5) or a camera resolution (Table 4). According to the subjective opinions of operators working at least 15 years as anesthesiologists, there is a concern that, in the case of a difficult intubation due to a slight oral dilation, the device will not go through the mouth. Moreover, as previously mentioned, the LCD screen in view of a rigid fixing does not enable full visualization on the early stages of entering the mouth. The operator has to lean over the patient to have an overview of the preliminary stage of a laryngoscope placement in the mouth.
- Vividtrac: While collecting references for this article we have not found extensive research reports indicating the positive effects of the use of this device. The single films demonstrating the efficacy of this device can be found on the manufacturer’s web pages. This device appeared to be very convenient to use according to our practical knowledge. In our opinion, its small size and structure fits very well to supply the patient with a difficult airway. The great possibility is that it can be connected to almost any device with a USB port. This device has an endoscopic lamp with a strong intensity (Table 2) and although it is not the strongest of all comparable devices, sometimes the observed tissues were overexposed when placing too close to the camera (Fig. 12), and also there is a tendency to move hue towards red which promotes the impression of congestion. However, this device has definitely the largest field of view in the horizontal axis. In our opinion, it affects positively the clinical aspects related to the use of this device. From our point of view, the disadvantage is that it is a disposable device, while at the same time due to a possibility of combining with other electronic devices it suits educational purposes well.

All compared devise are dedicated for adult patients. However, manufacturers prepare new versions of KingVision, McGrath Mac for children, and smaller size of C-Mac D-blade. Vividtrac so far is only in one size.

### Limitations

- A limitation of our study may be the fact that due to a lack of possibility to take a screenshot of the Kingvision and the Mac McGrath on the electronic media, we were not able to compare all the parameters of the other devices. The One size of the laryngoscope blade can be a disadvantage in McGrath MAC videolaryngoscope. Cmac and Kingvision devices have several different sizes of blades for adults and children. The measurement error is also influenced by the fact that the cameras are hidden inside the devices and it was impossible to measure the exact distance between the sensor and the distal blade tip.

## Conclusions

- In the following statement, the Cmac has better specifications than other devices in almost all examined aspects.
- The Vividtrac is definitely best suited to train students in the context of clinical practice in real-time due to the possibility of transferring the image on the big screen.
- It would be useful to reflect on possible improvements in particular devices that could eliminate the identified limitations in their use. It is worth to lead the next benchmarking and broaden this knowledge.

## References

[1] Chemsian RV, Bhananker S, Ramaiah R (2014). Videolaryngoscopy. Int J Crit Illn Inj Sci.

[2] Wayne MA, McDonnell M (2010). Comparison of traditional versus videolaryngoscopy in out-of-hospital tracheal intubation. Prehosp Emerg Care.

[3] Nouruzi-Sedeh P, Schumann M, Groeben H (2009). Laryngoscopy via Macintosh blade versus GlideScope: success rate and time for endotracheal intubation in untrained medical personnel. Anesthesiology.

[4] Sun DA, Warriner CB, Parsons DG, Klein R, Umedaly HS, Moult M (2005). The GlideScope video laryngoscope: randomized clinical trial in 200 patients. Br J Anaesth.

[5] Platts-Mills TF, Campagne D, Chinnock B, Snowden B, Glickman LT, Hendey GW (2009). A comparison of GlideScope videolaryngoscopy versus direct laryngoscopy intubation in the emergency department. Acad Emerg Med.

[6] Lim HC, Goh SH (2009). Utilization of a GlideScope videolaryngoscope for orotracheal intubations in different emergency airway management settings. Eur J Emerg Med..

[7] http://www.kingsystems.com/medical-devices-supplies-products/airway-management/video-laryngoscopes/.

[8] http://www.emsworld.com/product/10772836/karl-storz-endoscopy-america-inc-c-mac-pocket-monitor.

[9] http://creative.epsinternet.com/apps/mediabucket2/C-MAC-EW-hryb.pdf.

[10] Ray DC, Billington C, Kearns PK, Kirkbride R, Mackintosh K (2009). A comparison of McGrath and Macintosh laryngoscopes in novice users: a manikin study. Anaesthesia..

[11] Shippey B, Ray D, McKeown D (2007). Case series: the McGrath videolaryngoscope- an initial clinical evaluation. Can J Anesth.

[12] Shippey B, Ray D, McKeown D (2008). Use of McGrath videolaryngoscope in the management of difficult and failed tracheal intubation. Br J Anaesth.

[13] Shimada N, Hayashi K, Sugimoto K, Takahashi M, Niwa Y, Takeuchi M (2013). The KINGVISION: clinical assessment of performance in 50 patients. Masui.

[14] Hayashi K, Shimada N, Shiba J, Niwa Y, Takeuchi M (2014). A manikin study of the KingVision videolaryngoscope compared with Airwayscope. Masui..

[15] Healy D, Picton P, Morris M, Turner C (2012). Comparison of the glidescope, CMAC, storz DCI with the Macintosh laryngoscope during simulated difficult laryngoscopy: a manikin study. BMC Anesthesiol.

[16] Lipe DN, Lindstrom R, Tauferner D, Mitchell C, Moffett P (2014). Evaluation of Karl Storz CMAC Tip™ device versus traditional airway suction in a cadaver model. West J Emerg Med.

[17] Mutlak H, Rolle U, Rosskopf W, Schalk R, Zacharowski K, Meininger D, Byhahn C (2014). Comparison of the TruView infant EVO2 PCD™ and C-MAC video laryngoscopes with direct Macintosh laryngoscopy for routine tracheal intubation in infants with normal Airways. Clinics (Sao Paulo).

[18] Ng I, Hill AL, Williams DL, Lee K, Segal R (2012). Randomized controlled trial comparing the McGrath videolaryngoscope with the C-MAC videolaryngoscope in intubating adult patients with potential difficult airways. Br J Anaesth.

[19] Taylor AM, Peck M, Launcelott S, Hung OR, Law JA, MacQuarrie K, McKeen D, George RB, Ngan J. The McGrath® Series 5 videolaryngoscope vs the Macintoshlaryngoscope: a randomised, controlled trial in patients with a simulated difficult airway. Anaesthesia. 2013;68(2):142–7. doi:10.1111/anae.12075. 10.1111/anae.12075 23121470

[20] Gaszynski T (2014). Clinical experience with the C-Mac videolaryngoscope in morbidly obese patients. Anaesthesiol Inten Ther.

